# Identification of a novel *NPM1* mutation in acute myeloid leukemia

**DOI:** 10.1186/s40164-023-00449-4

**Published:** 2023-10-04

**Authors:** Yiyi Yao, Xiangjie Lin, Chen Wang, Ying Gu, Jie Jin, Yinghui Zhu, Huafeng Wang

**Affiliations:** 1 Department of Hematology, The First Affiliated Hospital, Zhejiang University School of Medicine, 79, Qingchun Road, Hangzhou, 310003 People’s Republic of China; 2 Zhejiang Provincial Key Laboratory of Hematopoietic Malignancy, Zhejiang University, Hangzhou, 310000 Zhejiang People’s Republic of China; 3 Zhejiang Provincial Clinical Research Center for Hematological Disorders, Hangzhou, 310000 Zhejiang People’s Republic of China; 4 Zhejiang University Cancer Center, Hangzhou, 310000 Zhejiang People’s Republic of China; 5 Institute of Genetics, Zhejiang University and Department of Genetics, Zhejiang University, School of Medicine, Hangzhou, 310058 Zhejiang People’s Republic of China; 6 Research Center for Translational Medicine, Shanghai East Hospital, Frontier Science Center for Stem Cell Research, Shanghai Key Laboratory of Signaling and Disease Research, School of Life Sciences and Technology, Tongji University, 1239 Siping Road, Shanghai, 200092 China

**Keywords:** *NPM1* mutation, Acute myeloid leukemia, Aberrant cytoplasmic dislocation, Nuclear export signal, Prognosis

## Abstract

Nucleophosmin (NPM1) is a widely expressed nucleocytoplasmic shuttling protein with prominent nucleolar localization. It is estimated that 25–35% of adult patients with acute myeloid leukemia (AML) carry *NPM1* mutations. The classic *NPM1* type A mutation occurs in exon 12, which accounts for 75–80% of adult patients with *NPM1*-mutated AML. It produces an additional leucine and valine-rich nuclear export signal (NES) at the C-terminus, and causes aberrant cytoplasmic dislocation of NPM1 protein. Notably, emerging evidence indicates that besides the classic type A mutation, rare mutants occurring in other exons may also lead to the imbalance of the nucleocytoplasmic shuttle of NPM1. Identification of novel non-type A mutants is crucial for the diagnosis, prognosis, risk stratification and disease monitoring of potential target populations. Here we reported a novel *NPM1* mutation in exon 5 identified from a de novo AML patient. Similar to the classic type A mutation, the exon 5 mutation had the NPM1 mutant bound to exportin-1 and directed the mutant into the cytoplasm by generating an additional NES sequence, resulting in aberrant cytoplasmic dislocation of NPM1 protein, which could be reversed by exportin-1 inhibitor leptomycin B. Our findings strongly support that besides the exon 12 mutation, the exon 5 mutant is another *NPM1* “born to be exported” mutant critical for leukemogenesis. Therefore, similar to the classic type A mutation, the identification of our novel *NPM1* mutation is beneficial for clinical laboratory diagnosis, genetic risk assessment and MRD monitoring.

**Supplementary Information:**

The online version contains supplementary material available at 10.1186/s40164-023-00449-4.

**To the Editor**,

Nucleophosmin (NPM1) is a ubiquitously expressed nucleocytoplasmic shuttling protein, with predominant nucleolar localization. *NPM1*-mutant acute myeloid leukemia (AML) accounts for 25–35% of adult AML patients, has been defined as a distinct AML entity in the 2022 World Health Organization (WHO) classification and is validated as a marker of measurable residual disease (MRD) [1, 2]. The majority of *NPM1* mutations reported affect exon 12 (classic type A mutation), accounting for 75–80% of adult *NPM1*-mutated AML cases, which generates an extra nuclear export signal (NES) in the C-terminus leading to aberrant cytoplasmic localization [3]. However, accumulating evidence suggests that other than the canonical type A mutation, some novel mutations identified in exons 9 and 11, may also result in NPM1 nucleocytoplasmic shuttling. (Additional file 1: Table S1) [4–7]. Recent large clinical studies reported that AML patients with *NPM1* mutations alone had a favorable outcome, compared with *NPM1* mutations combined with *FLT3-ITD* [8]. Moreover, the type A mutation was associated with a favorable prognosis, while some newly identified non-type A mutants were associated with poor clinical outcomes [2, 9]. Thus, identification of novel non-type A mutants is paramount for diagnosis, prognosis, risk stratification, and disease monitoring of potential target populations.

## Identification of a novel *NPM1* mutation located in exon 5

**Fig. 1:**
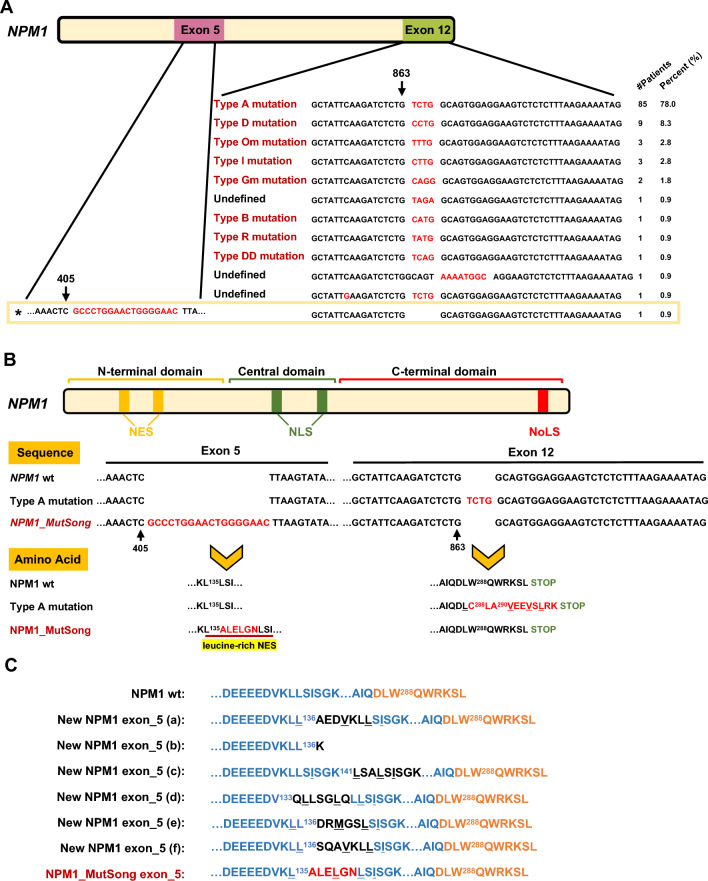
**A** Alignment of genomic sequences of the new *NPM1* exon 5 mutant (highlighted) and exon 12 mutants including canonical type A mutant and other rare mutants that identified in our AML patient cohort. The patient numbers and percentages of different mutants were also indicated. **B** Graphical representation and predicted protein sequences of *NPM1* wildtype, type A mutant and the new *NPM1* exon 5 mutant (*NPM1_MutSong*). The new generated amino acids are highlighted in red. **C** Alignment of predicted protein sequences of *NPM1* wildtype, reported exon 5 mutants and the new *NPM1* exon 5 mutant. For the new exon 5 mutant, the predicted NES motif is underlined and highlighted

In this study, somatic mutation analysis was performed on samples from 566 AML patients using the next-generation sequencing (NGS) with myeloid sequencing panel (Additional file 1: Table S2). *NPM1* mutations were identified in 109 AML cases, with four mutation types (A, D, Om, and I), which predominately affected exon 12, represented the vast majority of *NPM1* mutations: the type A mutation accounted for 78.0% of overall cases, while types D, Om, and I in total accounted for 13.8% (Fig. 1A). Importantly, we identified one AML patient with *NPM1* mutation located in exon 5. This patient was a 59-year-old female with de novo AML (Additional file 1: Table S3), with an 18-nucletide in-frame insertion at position 405 (c.405_406insGCCCTGGAACTGGGGAAC, named *NPM1_MutSong*) in the middle of exon 5. It generated a new NPM1 mutant protein, which was 6 aa longer than the wildtype one (p.135insALELGN), and contained a leucine-rich NES (Fig. 1B). The mutation was heterozygous for the patient [variant allele fraction (VAF), 11.9%]. Notably, different from all *NPM1* mutants in exons 9, 11, and 12 reported so far, the newly-identified exon 5 mutant had an additional leucine-rich NES inserted in the intermediate domain but retained the C-terminal functional NoLS. Similar findings were recently reported on *NPM1* mutants in exon 5 but at different positions (Fig. 1C) [10].

## The novel *NPM1* mutant exhibited aberrant cytoplasmic dislocation

**Fig. 2:**
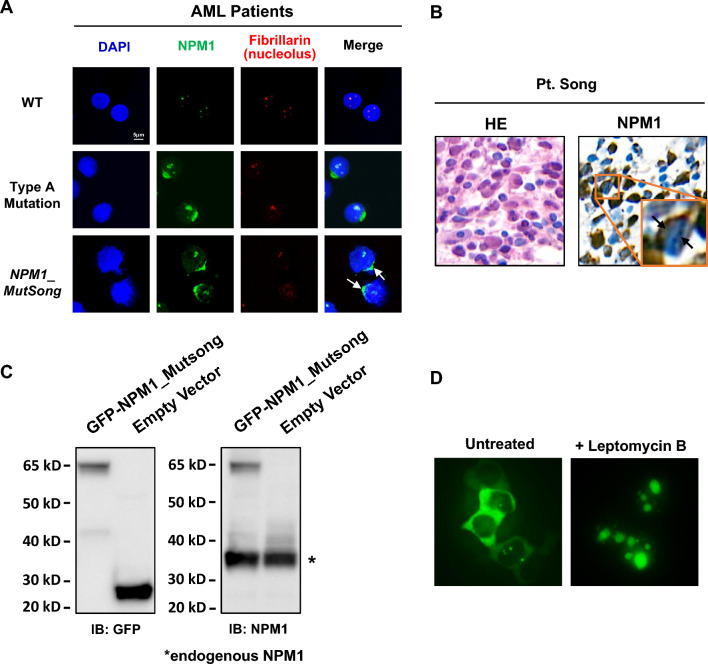
**A** Representative images show cellular localization of NPM1 (green) in blasts from typical AML patients, with *NPM1* wildtype, *NPM1* type A mutation and this new exon 5 mutation (*NPM1_MutSong*), respectively. DAPI was used for nuclei staining (blue). Fibrillarin was used for nucleolus staining. White arrows indicated the predominate cytoplasmic location of NPM1 in mutation *NPM1_MutSong.* Images were collected by Nikon eclipse Ti2 confocal microscope; magnification, × 600. **B** Immunohistochemical staining of bone marrow biopsies from Pt. Song with the new *NPM1* exon 5 mutation. Representative images show diffuse infiltration by leukemia blasts (hematoxylin and eosin [HE], left), with aberrant cytoplasmic localization of NPM1 (right, brown). Images were collected by Pannoramic 250 FLASH; magnification, × 630. **C** Western blot analysis with anti-GFP antibody (left, ~ 27 kD) or anti-NPM1 antibody (right, ~ 38 kD) both recognizing NPM1 proteins in HEK-293T cells overexpressing GFP-NPM1 exon 5 fusion protein (~ 61 kD). **D** HEK-293T cells overexpressing the new GFP-NPM1 fusion protein show the aberrant cytoplasmic subcellular location, concomitantly, with nucleolar location. Upon treatment with leptomycin B, NPM1 was re-localized in nucleus. Images were collected by Olympus IX71 confocal microscope; magnification, × 200

This *NPM1* mutant in exon 5 resulted in cytoplasmic localization detected by both confocal microscopy (Fig. 2A) and immunohistochemistry (IHC) (Fig. 2B) in the AML patient’s sample. Cytoplasmic dislocation was induced by the extra NES that partially impeded the NoLS driven nucleolar localization. HEK-293 T overexpressing the new GFP-NPM1 exon 5 fusion protein from patient Song (Fig. 2C) indicated the aberrant localization in the cytoplasm and partially in nucleoli (Fig. 2D, left). Moreover, NES-dependent cytoplasmic localization was inhibited by exportin-1 inhibitor leptomycin B (Fig. 2D, right), suggesting that the novel NES generated by the exon 5 mutant was functional and responsible for cytoplasmic localization.

## Clinical characteristics of the AML patient with the novel *NPM1* mutation

We then summarized the clinical characteristics of the AML patient (Pt. Song) harboring the novel *NPM1* exon 5 mutation (*NPM1_MutSong*) (Additional file 1: Table S3). Besides *NPM1* mutation, she also carried *WT1* (VAF, 13.1%), *IKZF1* (VAF, 17.8%), *JAK2* (VAF, 4.6%), and *NUP98* (VAF, 18.8%) mutations at the time of diagnosis. The patient was intravenously treated with daunorubicin 60 mg/m^2^ (days 1–3) plus cytarabine 100 mg/m^2^, intravenously (days 1–7) for induction, followed by 3 cycles of intermediate-dose cytarabine as consolidation. Then the patient received allogeneic hematopoietic stem cell transplantation (allo-HSCT). Unfortunately, Pt. Song died of severe pulmonary infection and viral encephalitis 14 months after the initial remission, 7 months after allo-HSCT.

The novel mutant exhibited several similar concomitant clinical features as type A mutation. Compared with commonly co-mutated genes such as *DNMT3A* and *TET2*, *NPM1* mutations are generally presented at lower VAFs [11]. Since mutations acquired later in disease development exhibited lower VAFs than earlier mutations [12], the moderate VAFs suggest that *NPM1* mutations may be a late event in leukemogenesis. In our patient, all the mutations showed moderate VAFs. Although our patient did not carry *FLT3-ITD* mutation, remarkably, she carried the *WT1* mutation. While the classic *NPM1* mutation alone is reportedly a favorable factor for AML with normal cytogenetics, sole *WT1* mutation has been reported to be associated with poor prognosis [13]. Interestingly, data from a large cohort of AML patients showed that the coexistence of *WT1* and *NPM1* mutations significantly altered the positive prognostic impact of *NPM1* mutations alone [13], underscoring the importance of more aggressive treatment for this subpopulation. Thus, our patient underwent allo-HSCT after initial remission, but unfortunately, ultimately died of severe pulmonary infection and viral encephalitis.

In this study, we reported a novel *NPM1* mutation in exon 5 in a de novo AML patient, which resulted in cytoplasmic dislocation of NPM1 protein. Our findings strongly support that besides the exon 12 mutation, the exon 5 mutant is another *NPM1* “born to be exported” mutant critical for leukemogenesis. Therefore, similar to the classic type A mutation, identification of our novel *NPM1* mutation is beneficial for clinical laboratory diagnosis, genetic risk assessment and MRD monitoring.

### Supplementary Information

**Additional file 1: Table S1.** Summary of reported rare *NPM1 *mutants and subcellular localization of mutated NPM1. **Table S2.** Next generation sequencing (NGS) gene panel. **Table S3.** Characteristics of the patient with AML carrying the *NPM1* exon 5 mutation.

## Data Availability

The datasets used and/or analyzed during the current study are available from the corresponding author on reasonable request.
