# Supplementary material for: Identification of a novel NPM1 mutation in acute myeloid leukemia
Source: Exp Hematol Oncol. 2023 Oct 4;12:87. doi: 10.1186/s40164-023-00449-4 (PMC10548603; doi:10.1186/s40164-023-00449-4)
Supplement: Supplementary file 1 — Additional file 1: Table S1. Summary of reported rare NPM1 mutants and subcellular localization of mutated NPM1. Table S2. Next generation sequencing (NGS) gene panel. Table S3. Characteristics of the patient with AML carrying the NPM1 exon 5 mutation. [file 40164_2023_449_MOESM1_ESM.docx]

**Identification of a Novel *NPM1* Mutation in Acute Myeloid Leukemia**

**Table S1**

**Summary of reported rare *NPM1* mutants and subcellular localization of mutated NPM1**

| **Mutation site** | **Exon** | **Confirmed/predicted localization of NPM1 protein** | **Reference** |
| --- | --- | --- | --- |
| c.755_771IndelTCCTTT | 9 | Cytoplasm | A R Mariano et al., 2006 ^1^ |
| c.806_807InsGGCGCCTA | 11 | Cytoplasm | E Albiero et al., 2007 ^2^ |
| c.805_806InsGCCCGGCT | 11 | Cytoplasm | A S Pitiot et al., 2007 ^3^ |
| # c.839_847delACCAAGAG | ACCAAGAG corresponding to the 3’-end of exon 11 and G corresponding to 5’-end of exon 12 | Nucleolus | Annalisa P et al., 2010 ^4^ |
| # c.864G>A | 12 | Nucleolus | Annalisa P et al., 2010 ^4^ |

**#:** Two *NPM1* mutations with different exons (exons 11 and 12) were identified in the same patient.

**Table S2**

**Next generation sequencing (NGS) gene panel**

| ABL1 | BCOR | CUX1 | FIP1L1 | IKZF1 | MET | NSD1 | PTEN | SMC3 | TP53 | RAD21 |
| --- | --- | --- | --- | --- | --- | --- | --- | --- | --- | --- |
| AKT1 | BCR | DNMT3A | FLT3 | JAK1 | MPL | NUP98 | PTPN11 | SRSF2 | U2AF1 | SMC1A |
| AKT2 | CALR | EP300 | GATA1 | JAK2 | MSH2 | PDGFRA | RARA | STAG2 | WT1 | KDM2B |
| AKT3 | CBL | ERG | GATA2 | JAK3 | MTOR | PDGFRB | RB1 | STAT3 | XPO1 | KDM5A |
| APC | CDKN2A | ETV6 | GATA3 | KIT | MYC | PHF6 | RUNX1 | STAT5A | ZRSR2 | KDM6A |
| ASXL1 | CEBPA | EWSR1 | HRAS | KMT2A | NF1 | PIK3CA | RUNX1T1 | STAT5B | PML | SRC |
| ATM | CSF1R | EZH2 | IDH1 | KMT2D | NPM1 | PRDM1 | SETBP1 | STAT6 | CBFB | BCL2 |
| ATRX | CSF3R | FGFR1 | IDH2 | KRAS | NRAS | PTCH1 | SF3B1 | TET2 | MYH11 | SMO |

**Table S3**

**Characteristics of the patient with AML carrying the *NPM1* exon 5 mutation**

| **Characteristics** | **Pt. Song** |
| --- | --- |
| Gender | Female |
| Age (years) | 59 |
| Diagnosis | De novo AML |
| FAB subtype | M0 |
| WBC (x 10^9^/L) | 2.6 |
| Hb (g/dl) | 95 |
| Plt x 10^9^/L | 45 |
| BM blasts (%) | 63 |
| Karyotype | 46,XX[11]/92,XXXX[1] |
| Cytogenetic risk | Intermediate |
| European Leukemia Net 2022 risk | Favorable |
| *WT1* (VAF%) | c.1071_1072insACCCAGCCGGGGA (13.1%) |
| *IKZF1* (VAF%) | c.1391 G>A (17.8%) |
| *JAK2* (VAF%) | c.2051 A>T (4.6%) |
| *NUP98* (VAF%) | c.1405_1406insGGCC (18.8%) |
| IHC | NPM1c+ |
| Treatment | DA + intermediate-dose cytarabine + BMT |
| Outcome | Died 15 months after diagnosis |

**WBC**, white blood cell; **Hb**, hemoglobin; **PLT**, platelet; **Pt.**, Patient; **VA**F, variant allele frequency; **IHC**, immunohistochemistry; **DA**, Cytarabine + daunorubicin; **BMT**, allogeneic bone marrow transplantation.

**Materials and Methods**

**Patient and sample collection**

Mononuclear cells were isolated from the patient’s bone marrow (BM) at diagnosis. The study protocol was approved by the institutional ethics committees, with the patient providing informed consent for clinical sample collection.

**Genomic analysis**

A DNA library was constructed using the KAPA Hyper DNA Library Prep Kit (KAPA Biosystems, MA, USA). Library concentration and quality were determined by the Qubit 3.0 system (Invitrogen, CA, USA) and Bioanalyzer 2100 (Agilent, Waldbronn, Germany). Hybridization capture was conducted with the biotinylated oligo probe and paired 150-bp reads on Illumina HiSeq 4000 (Illumina, CA, USA) were performed according to the manufacturer’s protocol. High quality reads were mapped to the human genome (hg19, GRCh37 Genome Reference Consortium Human Reference 37) using the Burrows-Wheeler Aligner and the publicly available SAM tools, Picard, and Genome Analysis Toolkit. Genomic alterations, including single nucleotide variants (SNVs), short and long insertions/deletions (indels), copy number variants (CNVs), and gene rearrangements and fusions were identified.

**Cell culture and transfection**

HEK-293T cells (ATCC, VA, USA) were cultured in DMEM medium supplemented with 10% fetal bovine serum (Gibico, NY, USA). The coding DNA sequence of the novel mutated *NPM1* was cloned into the pCDH vector to construct the overexpression vector. For ectopic expression of the GFP-NPM1 fusion protein, transient transfection was carried out using Calcium Phosphate Transfection Kit (C0508, Beyotime, Shanghai, China) according to the manufacturer’s instructions. After 48 hours incubation, cells were used for either immunofluorescence or Western Blot analysis. HEK-293T cells overexpressing the novel GFP-NPM1 fusion protein were then treated with the specific Crm1/XPO1 inhibitor leptomycin B (10 nM for 6 hours) (S1726, Beyotime, China) and were used to evaluate the NES dependence of subcellular localization by the Olympus IX71 fluorescence microscope (Olympus, Tokyo, Japan) (x 200 magnification).

**Western Blot**

GFP-NPM1 fusion protein was evaluated by western blot for expression, estimation of the molecular weight and certification of the reactivity with the anti-NPM1 specific antibody. NPM1-wild type HEK-293T cells transfected with empty vectors were used as the negative control. Approximately 2 x 10^6^ cells were lysed using RIPA buffer (89900, Thermo Fisher Scientific, MA, USA) supplemented with protease inhibitor and phosphatase inhibitor cocktail (1861280, Thermo Fisher Scientific, USA) on ice for 30 minutes. Samples were centrifuged at 12000 g for 15 minutes at 4°C and the supernatant protein concentration was determined using the BCA Protein Assay Kit (23228, Thermo Fisher Scientific, USA). Approximately 60 μg protein was loaded per well on 4-10% SDS-PAGE gel and transferred onto PVDF membranes (Millipore, MA, USA) preactivated with methanol. The membranes were blocked using 5% nonfat milk for 1 hour and incubated with primary antibodies, i.e. NPM1 monoclonal antibody (4F12A3, Proteintech, IL, USA) and GFP-Tag monoclonal antibody (YM3009, Immunoway, TX, USA) respectively, overnight at 4°C. After incubation, the membranes were washed with TBST and incubated with secondary antibodies for 1 hour at room temperature. Target proteins were then visualized using a ECL detection kit (34096, Thermo Fisher Scientific, USA) on ChemiDoc MP imaging system (Biorad, CA, USA).

**Immunofluorescent confocal analysis**

Cells were fixed in 4% paraformaldehyde. The cells were then blocked and permeabilized with PBS containing 5% BSA and 0.3 %Triton X-100 and incubated sequentially with: NPM1 monoclonal antibody (4F12A3, Proteintech, USA); Alexa Fluor 594 conjugated-anti-fibrillarin (ab203400, Abcam, Cambridge, UK); Alexa Fluor 488 conjugated-anti-mouse IgG (4408s, CST, MA, USA); and DAPI (C1006, Beyotime, China). Confocal microscopy and image acquisition were performed on Nikon eclipse Ti2 confocal microscope (Nikon, Tokyo, Japan) at 60X.

**Immunohistochemistry**

Immunohistochemical staining was performed on formalin-fixed paraffin-embedded human bone marrow samples. Briefly, the tissue sections were deparaffinized in xylene and rehydrated in ethanol, followed by antigen retrieval in citrate buffer (pH 6.0) and blocking endogenous peroxidase activity. The antibody/antigen interaction was detected with biotinylated anti-mouse antibody and mouse alkaline phosphatase-anti-alkaline phosphatase. Images were taken using Pannoramic 250 Flash III (3DHISTECH, Budapest, Hungary) at 63X and analyzed with the Pannoramic Viewer software.

**References**

1. Mariano AR, Colombo E, Luzi L, Martinelli P, Volorio S, Bernard L, et al. Cytoplasmic localization of NPM in myeloid leukemias is dictated by gain-of-function mutations that create a functional nuclear export signal. Oncogene. 2006; 25(31): 4376-80.
2. Albiero E, Madeo D, Bolli N, Giaretta I, Bona ED, Martelli MF, et al. Identification and functional characterization of a cytoplasmic nucleophosmin leukaemic mutant generated by a novel exon-11 NPM1 mutation. Leukemia. 2007; 21(5): 1099-103.
3. Pitiot AS, Santamaría I, García-Suárez O, Centeno I, Astudillo A, Rayón C, et al. A new type of NPM1 gene mutation in AML leading to a C-terminal truncated protein. Leukemia. 2007; 21(7): 1564-6.
4. Pianta A, Fabbro D, Damiani D, Tiribelli M, Fanin R, Franzoni A, et al. Two novel NPM1 mutations in a therapy-responder AML patient. Hematol Oncol. 2010; 28(3): 151-5.
